# Survival analysis of pneumoconiosis patients in Jiangsu Province from 1960 to 2024

**DOI:** 10.3389/fpubh.2025.1668318

**Published:** 2025-10-08

**Authors:** Ning Wang, Xinlei Chu, Lang Zhou, Yuan Zhao, Qianqian Gao, Yue Gao, Peng Zhou, Lei Han

**Affiliations:** ^1^School of Public Health, Nanjing Medical University, Nanjing, China; ^2^Institute of Occupational Disease Prevention, Jiangsu Provincial Center for Disease Control and Prevention, Nanjing, China

**Keywords:** occupational disease, pneumoconiosis, mortality rate, survival analysis, Cox regression analysis

## Abstract

**Introduction:**

Pneumoconiosis is recognized as a major occupational health burden worldwide, especially in developing countries where industrial dust exposure is prevalent. This study aims to provide evidence for the development of prevention and control strategies and the strengthening of patient support systems.

**Methods:**

Data for this study were sourced from the Jiangsu Province Pneumoconiosis Follow-up Online Reporting System. Survival analysis was performed employing life tables and the Kaplan–Meier method. The Cox proportional hazards regression models were deployed to identify factors influencing pneumoconiosis patients’ survival time.

**Results:**

A total of 18,064 patients were diagnosed with pneumoconiosis in Jiangsu Province between 1960 and 2024. Life table analysis revealed an overall cumulative survival rate of 40%. Both mortality and hazard ratios increased with advancing age. Patients with Stage III pneumoconiosis had a significantly lower survival rate compared with those in stages I and II (*p* < 0.05). The results showed that gender, stage at first diagnosis, age at first diagnosis, and disease progression were significant factors influencing survival time.

**Conclusion:**

Our findings highlight the importance of strengthening prevention and control measures targeting high-risk populations and key industries. Strategies should focus on early detection, timely diagnosis, and active intervention.

## 1 Introduction

Pneumoconiosis is a progressive and irreversible interstitial lung disease caused by the chronic inhalation of occupational dusts (1). It encompasses several major types, including silicosis, coal workers’ pneumoconiosis (CWP), and welders’ pneumoconiosis, all of which severely impair workers’ labor capacity and quality of life. Data from the Global Burden of Disease (GBD) 2021 study revealed a 62.5% increase in the number of incident pneumoconiosis cases between 1990 and 2021. Despite a decline in the age-standardized mortality rate, the burden of disability-adjusted life years (DALYs) from pneumoconiosis remains the highest among all occupational respiratory diseases considered in the GBD study, and the absolute number of deaths continues to rise (2). The current lack of effective therapeutic interventions and systematic rehabilitation management poses a significant public health and social security challenge regarding the survival outcomes of patients with pneumoconiosis (3).

As a country with a high incidence of pneumoconiosis, China has seen the number of pneumoconiosis cases consistently rank first among all occupational diseases since 2000. According to the “Bulletin on the Progress of Occupational Disease Prevention and Control” released by the National Health Commission in 2024, pneumoconiosis accounts for approximately 80% of all reported occupational diseases (4). The disease burden is particularly pronounced among males and migrant workers (5). In Jiangsu, a province characterized by a high degree of industrialization in manufacturing and mining, silicosis and CWP are the predominant types, collectively accounting for over 90% of all pneumoconiosis cases (6). Furthermore, active surveillance data from the province showed that out of 29,157 patients followed up between 2019 and 2022, a total of 11,918 deaths were recorded, reflecting the significant case-fatality rate of pneumoconiosis and its substantial, long-term demand on healthcare resources (7).

Jiangsu Province was selected as the focus of this study due to its unique position as one of China’s leading industrial and economic powerhouses. Unlike provinces dominated by a single resource, such as the coal-mining regions of Henan and Hubei, Jiangsu features a diverse and complex industrial structure encompassing not only mining but also extensive manufacturing (e.g., machinery, building materials, ceramics) and shipbuilding (8, 9). This mixed industrial layout results in a dual burden of both silicosis and coal workers’ pneumoconiosis (CWP), making it a representative model for understanding the epidemiological challenges in rapidly developing regions with multifaceted economies. Furthermore, this study leverages a high-quality, long-term surveillance database from Jiangsu, spanning over six decades (1960–2024) with a large cohort of over 18,000 patients. A systematic analysis of survival trends among pneumoconiosis patients can evaluate the real-world effectiveness of governmental dust control measures and occupational health policies, thereby providing evidence-based support for achieving the strategic goals of the “Healthy China 2030″ initiative. Moreover, updated survival prognosis data are crucial for optimizing follow-up management strategies, refining workers’ compensation insurance schemes, and improving the allocation of rehabilitation resources.

This study, based on the Jiangsu Provincial Pneumoconiosis Surveillance and Follow-up Database, aims to systematically analyze the epidemiological characteristics and survival outcomes of pneumoconiosis patients from 1960 to 2024. By identifying the prognostic factors influencing their survival time, we seek to provide scientific evidence and policy recommendations for the comprehensive prevention and control of occupational pneumoconiosis.

## 2 Study population and methods

### 2.1 Study population

The data for patients with pneumoconiosis were obtained from the Jiangsu Province Pneumoconiosis Follow-up Online Reporting System. We extracted all follow-up records of patients diagnosed in Jiangsu Province between 1960 and 2024. The cleaning and filtering process was conducted by our research team under the supervision of the Jiangsu Provincial Center for Disease Control and Prevention. Two independent reviewers cross-validated exclusions, with discrepancies resolved by a third senior researcher. After data cleaning, a total of 18,064 eligible patients were included in the final analysis. This study received ethical approval from the Ethics Review Committee of the Jiangsu Provincial Center for Disease Control and Prevention (Approval No. JSJK2022-B002-01).

### 2.2 Diagnostic criteria for pneumoconiosis

In this study, all diagnoses of pneumoconiosis were established by certified medical institutions for occupational disease diagnosis within Jiangsu Province. Each diagnosis was made in accordance with the national “Diagnostic Criteria of Occupational Pneumoconiosis” that was in effect at the time of the patient’s initial diagnosis (GBZ 70–2002, GBZ 70—2015). This process required a consensus diagnosis by a panel of at least three qualified radiologists, based on a comprehensive assessment of the patient’s occupational dust exposure history, clinical manifestations, epidemiological data, workplace environment, and findings from posteroanterior and lateral chest X-rays (10). Staging of pneumoconiosis was determined based on the “Radiological Diagnosis of Pneumoconiosis” standard (GB 5906—1997) and categorized into three stages: Stage I, Stage II, and Stage III. Disease progression could occur from Stage I to Stage II, Stage II to Stage III, or directly from Stage I to Stage III. For reference, the key criteria from the current GBZ 70–2015 standard are provided in Appendix S1.

### 2.3 Definitions of key variables and endpoints

In this study, the time of first diagnosis of pneumoconiosis was defined as the entry time for the survival analysis. The primary endpoint was death resulting from pneumoconiosis or its associated complications (e.g., tuberculosis, cor pulmonale), which constituted an event. The determination of the primary cause of death was based on official death certificates and medical records from the surveillance system. Associated complications were considered part of the event only if their diagnosis occurred after the initial diagnosis of pneumoconiosis. Cases were considered censored if the patient died from causes unrelated to pneumoconiosis, was lost to follow-up, or was still alive at the end of the observation period.

### 2.4 Statistical analysis

A patient database was established using Microsoft Excel 2016, and all statistical analyses were performed with SPSS 27.0 and R 4.2.3. Categorical variables were described as frequencies and percentages (%), and differences between groups were compared using the chi-square (*χ*^2^) test. The life table method was employed to calculate the survival rates for the entire study cohort over time. Kaplan–Meier survival curves were generated for subgroup analysis, and differences in survival distributions were assessed using the log-rank test. A Cox proportional hazards regression model was used to evaluate prognostic factors. The following seven potential prognostic factors were included as covariates in the univariable analysis: gender, industry type, duration of dust exposure, type of pneumoconiosis, stage at first diagnosis, age at first diagnosis, and disease progression. Variables with a *p*-value less than 0.05 in the univariable Cox regression analysis were included in the multivariable analysis. Subsequently, a multivariable Cox proportional hazards model was fitted to identify independent predictors of survival, and a Nomogram prediction model was constructed. All statistical tests were two-sided, and *p* < 0.05 was considered statistically significant.

## 3 Results

### 3.1 Inclusion of study population

**Figure 1:**
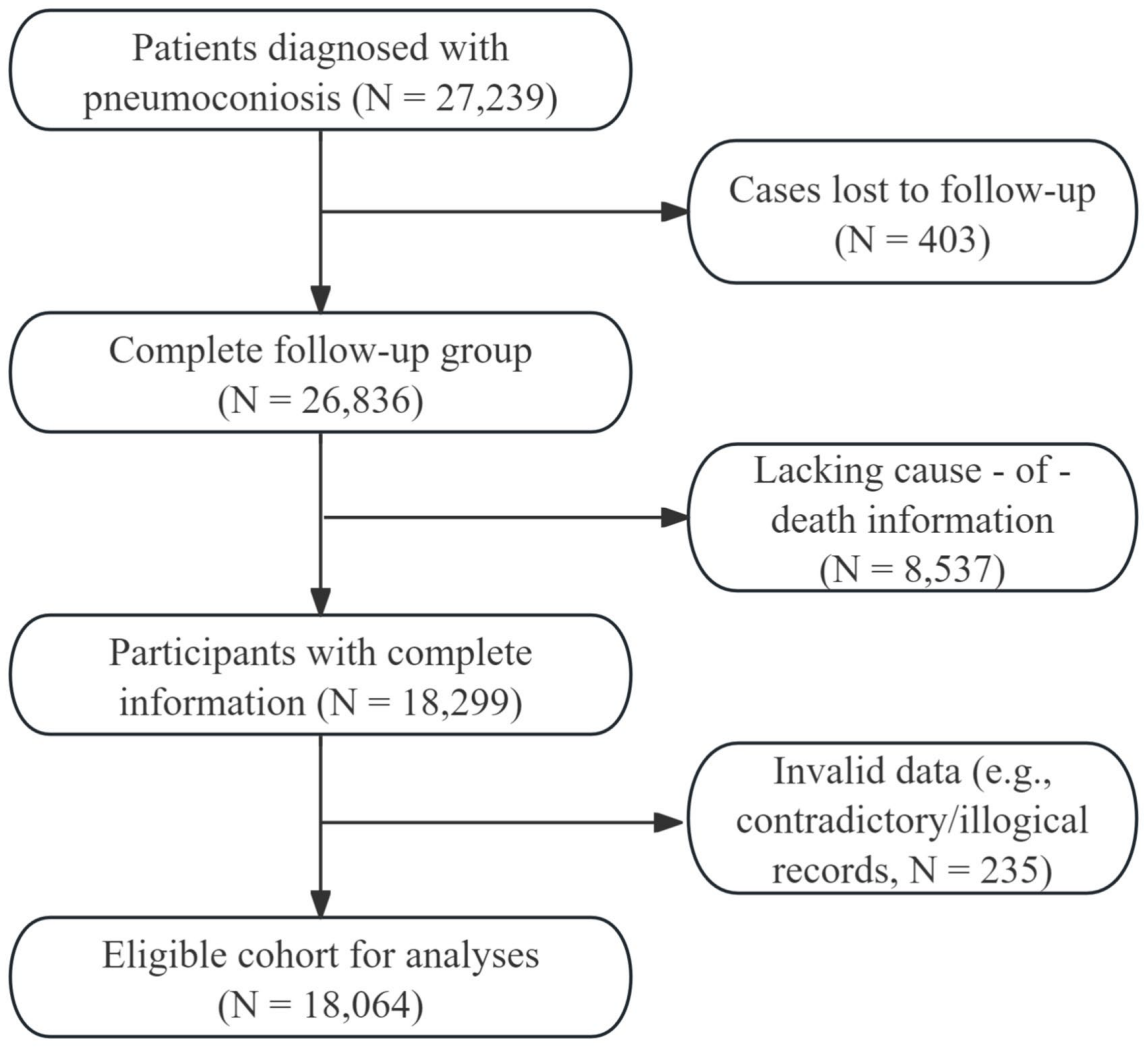
Flow chart of the study population inclusion.

Data for this study were sourced from the Jiangsu Province Pneumoconiosis Follow-up Online Reporting System, which initially comprised a cohort of 27,239 patients. To establish the final study cohort, we sequentially excluded individuals who were lost to follow-up (*n* = 403), those with missing data on key variables such as cause of death (*n* = 8,537), and cases with logical inconsistencies (*n* = 235), such as a dust-exposure duration longer than age or a date of death preceding the date of diagnosis. After this screening process, a total of 18,064 patients first diagnosed with pneumoconiosis in Jiangsu Province between 1960 and 2024 were included in the final analysis. The detailed flowchart of patient inclusion is presented in Figure 1.

### 3.2 Demographic and occupational characteristics

A total of 18,064 patients diagnosed with pneumoconiosis in Jiangsu Province between 1960 and 2024 were included in this study. Patients were stratified into three groups based on their pneumoconiosis stage at first diagnosis: 84.9% in Stage I (*n* = 15,338), 11.9% in Stage II (*n* = 2,158), and 3.2% in Stage III (*n* = 568). The detailed demographic and occupational characteristics are presented in Table 1. Across all stages, the prevalence was substantially higher in males at 93.4% (*n* = 16,871) than in females at 6.6% (*n* = 1,193). By disease type, silicosis was the most common form, accounting for 68.0% (*n* = 12,275) of cases, followed by CWP at 17.2% (*n* = 3,100). Regarding occupational distribution, mining was the most frequently reported industry, representing 49.8% (*n* = 9,000) of patients, followed by manufacturing at 25.6% (*n* = 4,625), and public administration, social security, and social organizations at 21.8% (*n* = 3,934). Notably, the largest proportion of patients, 44.8% (*n* = 8,098), were under 50 years of age at first diagnosis. During the follow-up period, the majority of patients, 92.8% (*n* = 16,763), remained at their initial diagnostic stage without apparent progression. Among those who progressed, 5.4% (*n* = 972) advanced from Stage I to II, 0.9% (*n* = 166) from Stage II to III, and another 0.9% (*n* = 169) directly from Stage I to III. Comparisons of all variables across the three pneumoconiosis stages revealed statistically significant differences (all *p* < 0.001).

**Table 1:** Demographic and occupational characteristics.

Variables	Stage I *N* = 15,338	Stage II *N* = 2,158	Stage III *N* = 568	*X* ^2^	*P*
Gender *n*(%)				14.391	<0.001
Male	14,369 (93.7%)	1975 (91.5%)	531 (93.5%)		
Female	969 (6.3%)	183 (8.5%)	37 (6.5%)		
Industries *n*(%)				474.191	<0.001
Mining	7,206 (47.0%)	1,471 (68.2%)	323 (56.9%)		
Manufacturing	3,963 (25.8%)	490 (22.7%)	172 (30.3%)		
Socialization^a^	3,705 (24.2%)	165 (7.6%)	64 (11.3%)		
Othres^b^	464 (3.0%)	32 (1.5%)	9 (1.6%)		
Dust exposure years *n*(%)				37.636	<0.001
≤10	5,543 (36.1%)	658 (30.5%)	208 (36.6%)		
11–20	5,048 (32.9%)	786 (36.4%)	204 (35.9%)		
21–30	3,695 (24.1%)	532 (24.7%)	111 (19.5%)		
>30	1,052 (6.9%)	182 (8.4%)	45 (7.9%)		
Type *n*(%)				178.862	<0.001
Silicosis	10,134 (66.1%)	1,663 (77.1%)	478 (84.2%)		
CWP^c^	2,811 (18.3%)	247 (11.4%)	42 (7.4%)		
Others^d^	2,393 (15.6%)	248 (11.5%)	48 (8.5%)		
Age at first diagnosis *n*(%)				107.651	<0.001
≤50	7,062 (46.0%)	859 (39.8%)	177 (31.2%)		
51–59	3,617 (23.6%)	674 (31.2%)	167 (29.4%)		
≥60	4,659 (30.4%)	625 (29.0%)	224 (39.4%)		

^a^Public Administration, Social Security and social organization; ^b^Other types of industries include: production and supply of electricity, heat, gas and water, construction, transportation, warehousing and postal services, resident services, repairs and other services; ^c^Coal workers’ pneumoconiosis; ^d^Other types of pneumoconiosis include: graphite pneumoconiosis, carbon black pneumoconiosis, asbestosis, talc pneumoconiosis, pottery worker’s pneumoconiosis, aluminosis, other pneumoconiosis, pneumoconiosis (unknown).

### 3.3 Survival rates and hazard ratios by age group

Table 2 presents the cumulative survival rates and corresponding hazard ratios for patients with pneumoconiosis across different age groups. For this analysis, patients were stratified into 10 groups based on 5-year age intervals. The probability of death from pneumoconiosis was estimated for each age group using adjusted observational data. The results demonstrated a clear trend: as age increased, the cumulative mortality rate progressively rose, ranging from 0.0003 to 0.3706. Concurrently, the hazard ratio also exhibited an increasing trend, escalating from 0.0003 to 0.0512. These findings suggest that advancing age is a significant determinant of mortality risk in patients with pneumoconiosis.

**Table 2:** Cumulative survival rate and probability of death in 18,064 patients with pneumoconiosis: grouping by age.

Age groups	Observed patients	Surviving patients	Died patients	Cumulatively observed patients	Corrected observed patients	Mortality rate	Survival rate	Cumulatively survival rate	Cumulatively mortality rate	Hazard rate
<35	5	5	0	18,064	18061.5	0.0000	1.0000	1.0000	0.0000	0.0000
35-	63	58	5	18,059	18,030	0.0003	0.9997	0.9997	0.0003	0.0003
40-	122	114	8	17,996	17,939	0.0004	0.9996	0.9993	0.0007	0.0004
45-	300	283	17	17,874	17732.5	0.0010	0.9990	0.9983	0.0017	0.0010
50-	713	668	45	17,574	17,240	0.0026	0.9974	0.9957	0.0043	0.0026
55-	1,103	987	116	16,861	16367.5	0.0071	0.9929	0.9887	0.0113	0.0071
60-	1,306	1,102	204	15,758	15,207	0.0134	0.9866	0.9754	0.0246	0.0135
65-	2,336	1928	408	14,452	13,488	0.0302	0.9698	0.9459	0.0541	0.0307
70-	4,635	4,133	502	12,116	10049.5	0.0500	0.9500	0.8986	0.1014	0.0512
≥75	7,481	6,163	1,318	7,481	4399.5	0.2996	0.7004	0.6294	0.3706	0.0000

### 3.4 Univariable survival analysis of factors influencing patient outcomes

**Figure 2:**
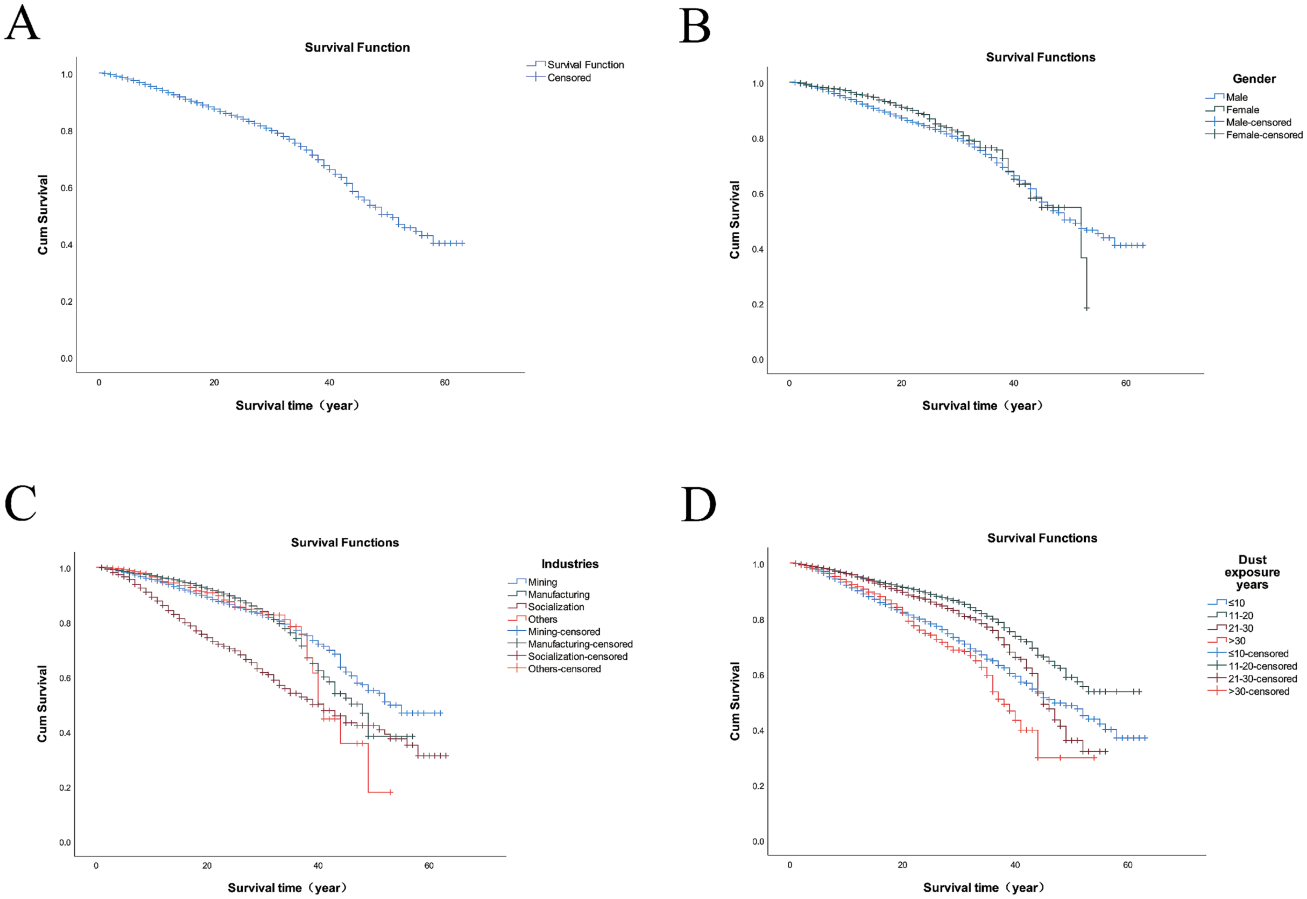
Survival curves of 18,064 pneumoconiosis patients across various cohorts in Jiangsu Province. The cohorts were categorized based on the following criteria: **(A)** the cumulative survival curve for the entire cohort; **(B)** the gender of pneumoconiosis; **(C)** the industries of pneumoconiosis; **(D)** the duration of dust exposure.

The survival probability of the 18,064 patients with pneumoconiosis diagnosed between 1960 and 2024 in Jiangsu Province was estimated using the Kaplan–Meier method. The survival time ranged from less than 1 year to a maximum of 63 years. The estimated survival rates at 10, 20, and 40 years post-diagnosis were 94, 87, and 65%, respectively, with an overall cumulative survival rate of 40%. The cumulative survival curve for the entire cohort is shown in Figure 2A.

#### 3.4.1 Effect of gender on survival

The median survival times for male and female patients were 51.1 years and 52.3 years, respectively. The log-rank test revealed a significant difference in survival distributions between genders (*χ*^2^ = 6.004, *p* = 0.014). The corresponding survival curves are presented in Figure 2B.

#### 3.4.2 Effect of industry on survival

Patients were categorized into four groups based on their industry: mining, manufacturing, public administration/social security/social organizations and others. The median survival times for these groups were 47.7, 43.7, 39.4, and 46.2 years, respectively. Patients in the public administration, social security, and social organizations sector had the shortest median survival time. A significant difference in survival was observed among the industry groups (log-rank *χ*^2^ = 471.144, *p* < 0.001), as depicted by the survival curves in Figure 2C.

#### 3.4.3 Effect of dust-exposure duration on survival

Patients were stratified into four groups based on their duration of dust exposure: ≤10 years, 11–20 years, 21–30 years, and >30 years. The survival rate for the >30 years exposure group was significantly lower than that of the other groups. The log-rank test confirmed a statistically significant difference in survival among these groups (log-rank *χ*^2^ = 279.066, *p* < 0.001). The survival curves are shown in Figure 2D.

#### 3.4.4 Effect of pneumoconiosis type on survival

**Figure 3:**
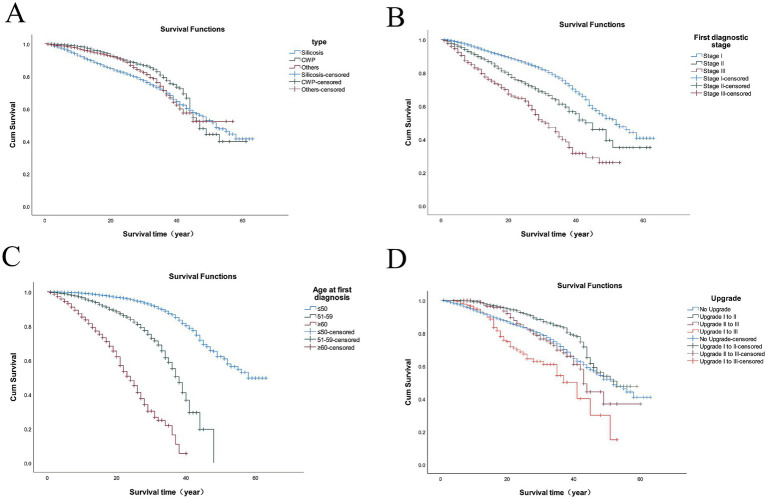
Survival curves of 18,064 pneumoconiosis patients across various cohorts in Jiangsu Province. The cohorts were categorized based on the following criteria: **(A)** the type of pneumoconiosis; **(B)** the first stage of diagnosis; **(C)** the age at first diagnosis; **(D)** the upgrade of pneumoconiosis.

Patients were classified into three groups by disease type: silicosis, CWP, and other pneumoconiosis. Their mean survival times were 45.7, 47.1, and 44.8 years, respectively. A statistically significant difference in survival time was found among the groups (log-rank *χ*^2^ = 76.229, *p* < 0.001). The corresponding survival curves are presented in Figure 3A.

#### 3.4.5 Effect of stage at first diagnosis on survival

As shown in Figure 3B, patients initially diagnosed at Stage I had a higher survival rate, with their survival curve positioned above those of Stage II and Stage III patients. Similarly, the survival curve for Stage II patients was higher than that for Stage III. The survival analysis confirmed significant differences in survival distributions among the diagnostic stages (log-rank *χ*^2^ = 363.355, *p* < 0.001).

#### 3.4.6 Effect of age at first diagnosis on survival

Patients were divided into three groups based on their age at first diagnosis: ≤50 years, 51–59 years, and ≥60 years. The mean survival times for these groups were 53.1, 35.4, and 23.4 years, respectively. The analysis revealed that the survival rate for patients diagnosed at ≥60 years of age was significantly lower than that of the other groups. The difference in survival among the three groups was statistically significant (log-rank *χ*^2^ = 3022.693, *p* < 0.001), with the results illustrated in Figure 3C.

#### 3.4.7 Effect of disease progression on survival

Patients were grouped based on disease progression: no progression, Stage I to II, Stage II to III, and Stage I directly to III. The survival curve for patients who progressed directly from Stage I to III was markedly lower than those of the other groups. A statistically significant difference in survival was observed among the four progression groups (log-rank *χ*^2^ = 70.688, *p* < 0.001), with the results illustrated in Figure 3D.

### 3.5 Multivariable Cox proportional hazards regression analysis

A Cox proportional hazards model was used to explore the effects of seven potential prognostic factors on the survival of patients with pneumoconiosis: gender, industry type, duration of dust exposure, type of pneumoconiosis, stage at first diagnosis, age at first diagnosis, and disease progression. Variables with *p* < 0.05 in the univariable analysis were subsequently included in a multivariable Cox regression model (Table 3).

**Table 3:** Cox regression analysis in 18,064 patients with pneumoconiosis.

Variables		Univariate Cox regression				Multivariate Cox regression			
		*β*	*P*	HR	95.0% CI	*β*	*P*	HR	95.0% CI
Gender	Male								
	Female	−0.202	0.015	0.817	0.694–0.961	−0.292	0.001	0.747	0.630–0.885
Industries	Mining		<0.001				<0.001		
	Manufacturing	−0.091	0.089	0.913	0.823–1.014	0.052	0.409	1.054	0.931–1.193
	Socialization	0.877	<0.001	2.403	2.196–2.629	0.295	<0.001	1.343	1.187–1.520
	Others	0.006	0.964	1.006	0.773–1.309	0.106	0.434	1.112	0.852–1.451
Dust exposure time /(years)	≤10		<0.001				0.472		
	11–20	−0.702	<0.001	0.495	0.450–0.546	−0.057	0.333	0.945	0.842–1.060
	21–30	−0.473	<0.001	0.623	0.563–0.689	−0.099	0.124	0.906	0.799–1.027
	>30	0.114	0.099	1.120	0.979–1.282	−0.036	0.649	0.964	0.824–1.128
Types	Silicosis		<0.001				<0.001		
	CWP	−0.581	<0.001	0.559	0.500–0.626	−0.291	0.000	0.747	0.661–0.845
	Others	−0.396	<0.001	0.673	0.592–0.766	0.187	0.016	1.206	1.035–1.405
First diagnostic stage	Stage I		<0.001				<0.001		
	Stage II	0.651	<0.001	1.917	1.735–2.119	1.315	<0.001	3.725	3.307–4.194
	Stage III	1.193	<0.001	3.296	2.806–3.872	2.628	<0.001	13.851	12.265–15.643
First diagnostic age	≤50		<0.001				<0.001		
	51–59	1.372	<0.001	3.943	3.513–4.425	0.610	<0.001	1.840	1.648–2.054
	≥60	2.727	<0.001	15.289	13.638–17.141	0.985	<0.001	2.677	2.273–3.154
Upgrade	No Upgrade		<0.001				0.018		
	Upgrade I to II	−0.558	<0.001	0.573	0.483–0.678	−0.108	0.221	0.897	0.754–1.067
	Upgrade II to III	0.019	0.903	1.019	0.753–1.379	0.345	0.027	1.411	1.040–1.916
	Upgrade I to III	0.648	<0.001	1.912	1.470–2.487	0.250	0.072	1.284	0.973–1.695

CI, confidence interval; HR, hazard ratio.

The results of the multivariable analysis indicated that gender, industry (specifically, public administration, social security, and social organizations), type of pneumoconiosis, stage at first diagnosis, age at first diagnosis, and disease progression were all significantly associated with mortality risk (all *p* < 0.05). In contrast, the duration of dust exposure was not found to be a significant predictor in the multivariable model (*p* > 0.05). Among all significant factors, being diagnosed at Stage III was the strongest predictor of mortality (HR = 13.851, 95% CI: 12.265–15.643), followed by a diagnosis at Stage II (HR = 3.725, 95% CI: 3.307–4.194) and an age at diagnosis of ≥60 years (HR = 2.677, 95% CI: 2.273–3.154). Other significant risk factors included an age at diagnosis of 51–59 years (HR = 1.840, 95% CI: 1.648–2.054), progression from Stage II to III (HR = 1.411, 95% CI: 1.040–1.916), employment in the public administration, social security, and social organizations sector (HR = 1.343, 95% CI: 1.187–1.520), and diagnosis with other types of pneumoconiosis (HR = 1.206, 95% CI: 1.035–1.405).

**Figure 4:**
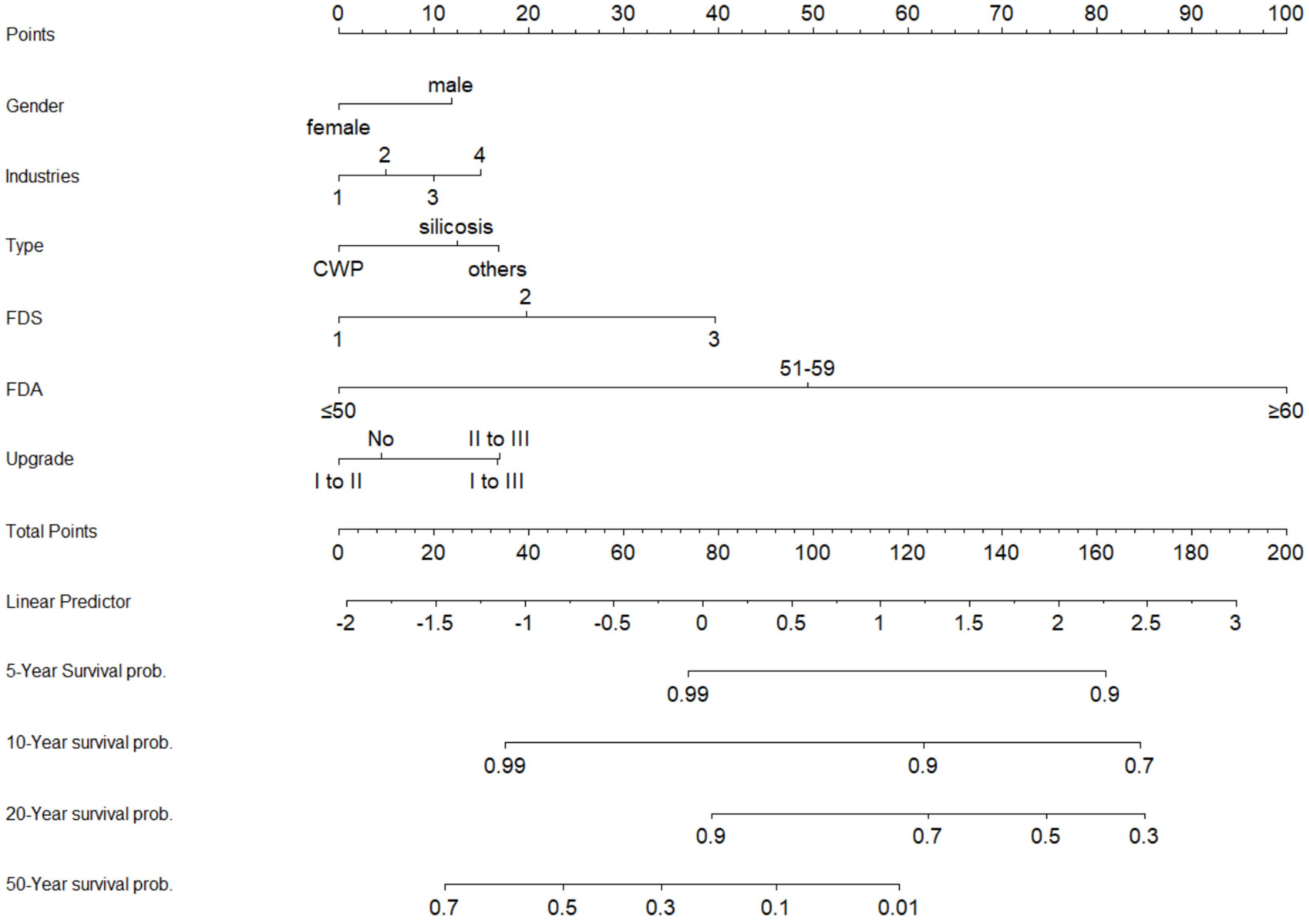
The nomogram of multivariate Cox proportional hazards model. Industries: 1, mining; 2, manufacturing; 3, organization; 4, others; FDS, first diagnostic stage; FDA, first diagnostic age.

To provide a more intuitive visualization of the combined effects of multiple factors on patient survival, a nomogram was constructed based on the multivariable Cox proportional hazards regression model (Figure 4). This nomogram integrates the scores of significant risk factors to predict the 5, 10, 20, and 50-year survival probabilities for individual patients. The results indicated that the stage at first diagnosis, age at first diagnosis, and disease progression demonstrated a strong predictive performance in the model.

## 4 Discussion

Pneumoconiosis is one of the most prevalent occupational lung diseases worldwide, affecting millions of workers in high-risk industries. This study, conducted in Jiangsu, a major industrial province in China, systematically evaluated the survival outcomes and associated prognostic factors based on a large cohort of 18,064 confirmed cases of pneumoconiosis. Our principal findings indicate that patients diagnosed at Stage III have a significantly shorter survival time compared to those at Stage I and II. Furthermore, industry type, age at first diagnosis, stage at first diagnosis, and disease progression were all found to be significantly associated with patient survival.

Previous studies have established that the regional epidemiological characteristics of occupational pneumoconiosis are highly correlated with local industrial structures (6). Globally, the three most common types of pneumoconiosis are asbestosis, silicosis, and CWP (11). Silicosis is predominant in developing countries, whereas asbestosis is more common in developed nations (12). In China, data reported from 2015 to 2020, both nationally and provincially, indicate that CWP and silicosis are the two most frequently reported types among new cases (13, 14). Consistent with these trends, our study identified silicosis and CWP as the primary types of pneumoconiosis in Jiangsu Province, a finding attributable to the high concentration of high-risk industries such as nonferrous metal, metallurgy, and coal mining (6). This distribution pattern is similar to that observed in other Chinese provinces, including Guangdong, Xinjiang Uygur Autonomous Region, and Gansu (15–17). For instance, silicosis is the principal type in Guangdong, followed by welders’ pneumoconiosis and other forms.

Regarding the distribution of disease stages, our study found that Stage I patients constituted the vast majority (84.9%), with progressively smaller proportions in Stage II and Stage III. This trend aligns with the findings of Yang et al., who investigated staging characteristics among pneumoconiosis patients across multiple provinces in China (18). Notably, our observation that Stage III patients have a significantly shorter survival time compared to those in Stages I and II is also consistent with previous research (19). This reinforces the conclusion that advanced-stage pneumoconiosis is associated with a markedly poor prognosis (20). The development of pneumoconiosis is a progressive process; as pathological damage to lung tissue worsens, the disease advances through stages, ultimately leading to irreversible impairment of pulmonary function and severely impacting patient survival (21). Therefore, early detection, timely intervention, and effective control of disease progression are of paramount importance for improving the quality of life and prolonging the survival of patients with pneumonoconiosis.

The life table analysis revealed that patient survival rates decreased with advancing age, while the corresponding hazard rates increased, suggesting that diagnosis at an older age is a significant risk factor for a poor prognosis in pneumonoconiosis. This finding was further substantiated by the stratified Kaplan–Meier survival analysis, which showed that the survival curves for patients in high-exposure, older-age, and Stage III groups were significantly lower than those for their counterparts in low-exposure, younger, and early-stage (I and II) groups (all *p* < 0.001). These results indicate that both prolonged dust exposure and delayed diagnosis have a detrimental impact on patient survival.

Our Cox regression analysis identified industry type (specifically, public administration, social security, and social organizations), age at first diagnosis, stage at first diagnosis, and disease progression as significant independent predictors of survival in patients with pneumonoconiosis. Although duration of dust exposure was statistically significant in the univariable analysis, its effect became non-significant in the multivariable model after adjusting for other variables. This may be attributable to a degree of collinearity with the age at diagnosis, as the latter might have partially mediated the effect of exposure duration in the model. Indeed, previous research has also noted that the prognostic effect of dust exposure duration may be attenuated after adjusting for age at diagnosis, suggesting a complex interplay between these two factors (22). Regarding the influence of industry, our finding that patients employed in the public administration, social security, and social organizations sector had a relatively lower survival rate is intriguing. A plausible explanation is that these individuals may have had prolonged prior engagement in high-exposure occupations (e.g., mining, manufacturing) before transitioning to these non-dust-exposed roles. Due to the long latency period of pneumoconiosis, their diagnosis was often significantly delayed, occurring years after they had changed jobs. This delayed diagnosis, typically at an older age, adversely affects their prognosis and explains the observed higher mortality risk in this cohort (23). Furthermore, the age at first diagnosis also impacts patient prognosis. Younger patients at the time of diagnosis typically have a better immune status and physical function, which may be more conducive to slowing disease progression and improving survival probability (24).

Patients initially diagnosed at Stage II and Stage III exhibited substantially elevated mortality risks, with hazard ratios of 3.725 and 13.851, respectively. This underscores that as the disease advances, the increasing severity of pulmonary fibrosis leads to further deterioration of lung function, which significantly impairs both quality of life and survival time. Our findings demonstrate that an adverse prognosis is associated not only with being diagnosed at an advanced stage (II or III) but also with any disease progression observed during follow-up. Therefore, it is imperative to strengthen regular health surveillance for high-risk populations to facilitate the early identification of pneumoconiosis progression. Prompt and timely intervention is crucial to delay disease exacerbation, thereby improving therapeutic outcomes and life expectancy for patients.

This study has several strengths, including its large sample size of 18,064 confirmed pneumoconiosis cases, a long follow-up period spanning from 1960 to 2024, and comprehensive data coverage in Jiangsu Province. These advantages allow for a systematic evaluation of survival outcomes and their associated factors. However, certain limitations should be acknowledged. First, despite continuous updates to medical records through annual follow-ups, the retrospective nature of this study makes it susceptible to information bias. Second, our cause-of-death classification relied on the textual diagnosis from death certificates rather than on standardized International Classification of Diseases (ICD) codes. This was due to the lack of systematic coding in the historical surveillance data, which may introduce some variability. Future studies would benefit from prospectively collected data with harmonized ICD coding. Third, although our regression models included several key variables (such as industry type, duration of dust exposure, age and stage at diagnosis, and disease progression), residual confounding from unmeasured factors, such as differences in personal protective equipment use, the presence of industrial ventilation and smoking history, may still exist. Furthermore, our study was limited by the use of exposure duration as a proxy for dust exposure, as historical dust concentration data were unavailable to calculate a more precise cumulative exposure index. This precluded a more detailed dose–response analysis and represents a common challenge in retrospective occupational epidemiology.

Future research should incorporate occupational trajectory data to further investigate the impact of dust exposure interruption and job adjustments on the prognosis of pneumoconiosis. Concurrently, long-term health surveillance for workers who have changed jobs should be intensified, and efforts to minimize loss to follow-up should be enhanced. Such measures will improve the continuity and scientific rigor of future studies, thereby providing more robust evidence for the early screening, prompt diagnosis, and precision intervention of pneumoconiosis.

In addition, our results highlight the necessity of tailoring preventive strategies to different industrial contexts. In the mining industry, strengthening underground ventilation, adopting wet drilling techniques, and utilizing efficient dust control equipment, together with real-time monitoring of workplace dust levels, are critical to reducing exposure (25). In the manufacturing sector, particularly in machinery, building materials, and ceramics, process improvements such as enclosure, automation, and job rotation can effectively minimize direct and prolonged dust contact (26). For construction and shipbuilding, local exhaust ventilation and wet suppression during high-dust operations (e.g., cutting, grinding, sandblasting), combined with strict enforcement of personal protective equipment use, are essential (27). Across all industries, the establishment of regular health surveillance, early screening, and occupational health training is equally important to enhance workers’ awareness and self-protection. Taken together, a comprehensive prevention framework that integrates engineering, administrative, and personal protective measures provides an effective pathway to reducing pneumoconiosis incidence and improving long-term survival among high-risk populations.

## 5 Conclusion

This large-scale retrospective cohort study of 18,064 patients in Jiangsu Province from 1960 to 2024 systematically evaluated the key determinants of survival in pneumoconiosis. Our findings underscore the multifactorial nature of patient survival, with age at first diagnosis, clinical stage, and disease progression emerging as the most critical prognostic factors. Given that pneumoconiosis is an irreversible, progressive disease with no curative treatment, it is essential to reinforce long-term follow-up management and comprehensive control strategies. Such efforts are paramount to slowing disease progression, thereby ultimately improving both the quality of life and survival outcomes for patients.

## Data Availability

The datasets presented in this article are not readily available because patient privacy is involved. Requests to access the datasets should be directed to hanlei_jscdc@163.com.
