# Supplementary material for: Survival analysis of pneumoconiosis patients in Jiangsu Province from 1960 to 2024
Source: Front Public Health. 2025 Oct 8;13:1668318. doi: 10.3389/fpubh.2025.1668318 (PMC12540365; doi:10.3389/fpubh.2025.1668318)
Supplement: Supplementary file 1 [file Data_Sheet_1.PDF]

GBZ

National Occupational Health Standards of the People's Republic of China

GBZ 70—2015

Replacing GBZ 70-2009

Diagnosis of Occupational Pneumoconiosis

Diagnosis of Occupational Pneumoconiosis

2015-12-15 Issued 2016-05-01 Implemented

National Health and Family Planning Commission of the People's Republic of China issued

Preface

This standard is formulated in accordance with the "Occupational Disease Prevention and Control Law of the People's Republic of China".

Chapter 6 of this standard is recommendatory, while the rest are mandatory.

This standard is drafted in accordance with the rules given in GB/T 1.1-2009.

This standard replaces GBZ 70-2009 "Diagnostic Criteria for Pneumoconiosis".

The main modifications compared to GBZ 70-2009 are as follows:

——Modified the standard name;

——Added "Terms and Definitions";

——Eliminate the "observation target";

——Add staging criteria for pleural lesions in individuals exposed to asbestos dust in the diagnostic staging of X -ray chest radiographs;

——Added requirements for the optical density below the diaphragm in digital radiography chest images in C.1.3;

——In D. 3, the number of whole-lung images increased to 19.

——Change the name of Appendix E to "Technical Requirements for High Kilovoltage Chest Radiography X -ray";

——Added Appendix F Technical Requirements for Digital Radiography Chest X-rays.

The drafting organization responsible for this standard: National Institute of Occupational Health and Poison Control, Chinese Center for Disease Control and Prevention.

Participating drafting units of this standard: Zhejiang Academy of Medical Sciences, China Coal General Hospital, Shenzhen Occupational Disease Prevention and Treatment Hospital, Shanxi Jincheng Anthracite Mining Group Co., Ltd. General Hospital, Shanghai Pulmonary Hospital, Guangxi Zhuang Autonomous Region Occupational Disease Prevention and Treatment Research Institute, Zhejiang Changguang (Group) Co., Ltd. Occupational Disease Prevention and Treatment Institute.

The main drafters of this standard: Yu Chen, Li Dehong, Zhang Xing, Chen Junqiang, Zhao Ruifeng, Shao Renchao, Li Baoping, Li Zhimin, Mao Ling, Zhang Zhenming, Zhu Qiuhong, Li Shufeng, Li Haixue, Li Yiqi, Liu Jianxin, Luo Jun, Shi Jin, Li Zhongxue, Qi Fang.

The release history of the standards replaced by this standard is as follows:

—GB 5906—1986;

—GB 5906—1997;

—GBZ 70—2002;

—GBZ 70—2009.

Diagnosis of Occupational Pneumoconiosis

1 Scope

This standard specifies the diagnostic principles for occupational pneumoconiosis (hereinafter referred to as pneumoconiosis), the diagnostic staging of pneumoconiosis based on X-ray chest films, and the principles for management.

This standard applies to the diagnosis of various types of pneumoconiosis listed in the "Classification and Catalog of Occupational Diseases" promulgated by the state, including silicosis, coal workers' pneumoconiosis, graphite pneumoconiosis, carbon black pneumoconiosis, asbestosis, talc pneumoconiosis, cement pneumoconiosis, mica pneumoconiosis, pottery workers' pneumoconiosis, aluminum pneumoconiosis, welders' pneumoconiosis, foundry workers' pneumoconiosis, and other pneumoconioses.

2 Normative References

The following documents are indispensable for the application of this document. For dated references, only the edition cited applies. For undated references, the latest edition (including any amendments) applies.

GB/T 16180 Assessment of Work Ability—Classification of Disability Caused by Work-Related Injuries and Occupational Diseases

3 Terms and Definitions

The following terms and definitions apply to this document.

3.1 Pneumoconiosis

A disease primarily characterized by diffuse pulmonary fibrosis caused by long-term inhalation of productive mineral dust during occupational activities and its retention in the lungs.

3.2 Small Opacity Small Shadows

On a chest X -ray, shadows within the lung fields with a diameter or width not exceeding 10 mm. Small shadows are categorized into two types based on their morphology: round and irregular.

3.3 Profusion Density

The number of small shadows within a certain range. The density is divided into 4 major levels, each of which is further divided into 3 minor levels, resulting in a classification system of 4 major levels and 12 minor levels.

3.4 Large Opacity Shadows

On a chest X -ray, a shadow within the lung field with a diameter or width greater than 10 mm.

3.5 Small Opacity Aggregation

On the X -ray chest film, there is a noticeable increase in localized small shadows clustered within the lung fields, but they have not yet coalesced into large shadows.

3.6 Pleural Plaque

On a chest X -ray, localized pleural thickening with a thickness greater than 5 mm or localized calcified pleural plaques appearing in the lung fields, except in the apical regions and costophrenic angles. It is generally caused by prolonged exposure to asbestos dust.

3.7 Zone of Lung

On the chest X -ray, the vertical distance from the lung apex to the diaphragm is divided into three equal parts. Horizontal lines at these division points separate the left and right lung fields into upper, middle, and lower lung zones, totaling six lung zones bilaterally.

4 Diagnostic Principles

Based on a reliable history of exposure to productive mineral dust, the diagnosis is primarily established by technically qualified high-kilovoltage X -ray or digital radiography (DR) posteroanterior chest radiographs. This is combined with workplace occupational hygiene data, pneumoconiosis epidemiological survey materials, and occupational health surveillance records. Clinical manifestations and laboratory tests are referenced, and other similar pulmonary diseases are excluded. The diagnosis is then confirmed by comparison with standard diagnostic radiographs for pneumoconiosis.

If the clinical manifestations and laboratory findings of a worker meet the characteristics of pneumoconiosis, and there is no evidence to negate the inevitable connection with dust exposure, the case should be diagnosed as pneumoconiosis.

5 Diagnostic Staging

5.1 Stage I Pneumoconiosis

Any of the following manifestations:

a) There are small opacities with an overall profusion of category 1, distributed over at least two lung zones;

b) Exposure to asbestos dust, with overall density grade 1 small opacities, distributed in only one lung zone, accompanied by pleural plaques;

c) Exposure to asbestos dust, with an overall small opacity profusion of 0, but at least two lung zones showing a small opacity profusion of 0/1, accompanied by pleural plaques.

5.2 Stage II Pneumoconiosis

Any of the following manifestations:

a) There are small opacities with an overall profusion category of 2, distributed over more than 4 lung zones;

b) There are small opacities with an overall profusion of category 3, distributed over up to 4 lung zones;

c) Exposure to asbestos dust, with small opacities of overall profusion category 1, distributed over more than 4 lung zones, accompanied by pleural plaques that have extended to involve part of the cardiac border or diaphragmatic surface;

d) Exposure to asbestos dust, with small opacities of overall profusion category 2, distributed over at least four lung zones, accompanied by pleural plaques that have extended to involve part of the cardiac border or diaphragmatic surface.

5.3 Pneumoconiosis Staging

Any of the following manifestations:

a) There is a large shadow with a major axis not less than 20 mm and a minor axis greater than 10 mm;

b) There are small opacities with an overall profusion of category 3, distributed over more than four lung zones and with aggregation of small opacities;

c) Presence of small opacities with an overall profusion category of 3, distributed over more than four lung zones, along with large opacities;

d) Exposure to asbestos dust, with overall small opacity profusion grade 3, distributed over more than four lung zones, accompanied by single or multiple pleural plaques on one or both sides, the sum of whose lengths exceeds half the length of one side of the chest wall or involves the cardiac border causing partial blurring.

6 Handling Principles

6.1 Treatment Principles

Patients with pneumoconiosis should promptly cease exposure to dust and undergo comprehensive treatment based on their condition. They should actively prevent and treat tuberculosis and other complications to alleviate clinical symptoms, slow disease progression, prolong life expectancy, and improve quality of life.

6.2 Other Processing

If a work capacity assessment is required, it shall be handled in accordance with GB/T 16180.

7 Instructions for Proper Use of This Standard

See Appendix A.

8 Determination of Small Shadow Patterns, Density, and Distribution Range, with Additional Symbols

See Appendix B.

9 Chest Radiograph Quality and Quality Assessment

See Appendix C.

10 Radiographic Diagnostic Criteria for Pneumoconiosis Standard Films

See Appendix D.

11 Technical Requirements for High Kilovoltage Chest Radiography

See Appendix E.

12 Technical Requirements for Digital Chest Radiography

See Appendix F.

13 Requirements for Reading Pneumoconiosis Diagnostic Films

See Appendix G.

Appendix A (Informative) Guidelines for the Proper Use of This Standard

A.1 Key Diagnostic Points Explanation

A history of exposure to productive mineral dust is a fundamental condition for diagnosing pneumoconiosis, including details such as the work unit, job position, start and end times of exposure to productive dust during different periods, and the name of the dust involved. If the employer, despite being urged by the work safety regulatory authority, still fails to provide workplace dust monitoring results, occupational health surveillance records, or provides incomplete information, the diagnosis should be made by integrating the worker's clinical manifestations, auxiliary examination results, occupational history, and dust exposure history, while also considering the worker's own account and routine supervision and inspection information provided by the work safety regulatory authority.

The posteroanterior chest radiograph is the primary basis for diagnosis. The quality and evaluation of chest radiographs are detailed in Appendix C. The technical requirements for high-kilovoltage chest radiography and digital chest radiography are specified in Appendix E and Appendix F, respectively.

The content of workplace occupational hygiene investigations mainly includes the nature of dust exposure, the content of free silica in the dust, the dispersion degree of dust, the detection and monitoring results of dust concentration, dust control and suppression facilities in the workplace, and the status of personal protective equipment, to assess the level of exposure and cumulative exposure dose.

Epidemiological data on pneumoconiosis primarily refer to the historical incidence and prevalence of the disease within the enterprise.

Although patients with pneumoconiosis may exhibit varying degrees of respiratory symptoms, signs, and certain abnormal laboratory findings, none of these are specific. Therefore, they can only serve as references for the diagnosis of pneumoconiosis. The focus of clinical and laboratory examinations is to conduct differential diagnosis, aiming to exclude other pulmonary diseases that present with X -ray chest images similar to those of pneumoconiosis.

A.2 Dynamic Observation of Chest X-rays

The radiological changes in chest X-rays of pneumoconiosis are a gradual process. Dynamic series of chest X-rays can systematically observe the evolution of lesions, more accurately determine the nature of small opacities, and provide more reliable evidence for diagnosis. Therefore, in principle, a confirmed diagnosis can only be made with two or more dynamic chest X-rays taken at intervals of more than six months. However, under special circumstances, if there is a reliable history of exposure to productive inorganic dust and supporting occupational hygiene data, along with typical pneumoconiosis X-ray chest manifestations and clear clinical data excluding other diseases, a diagnosis may also be considered.

A.3 Expression of Pneumoconiosis Diagnosis Conclusion

The diagnosis of pneumoconiosis should be stated as “Occupational + specific name of pneumoconiosis + stage,” such as Occupational Silicosis Stage I, Occupational Coal Workers’ Pneumoconiosis Stage II, etc. For those not diagnosed with pneumoconiosis, it should be stated as “No Pneumoconiosis.”

Appendix B (Normative Appendix) Determination of Small Opacity Shape, Concentration, Distribution Range, and Additional Symbols

B.1 Small Shadows

B.1.1 Morphology and Size

B.1.1.1 Small Rounded Shadows

Using the English letters p, q, r to denote:

——p: The maximum diameter shall not exceed 1.5 mm;

——q: Diameter greater than 1.5 mm, not exceeding 3 mm;

—— r : Diameter greater than 3 mm , not exceeding 10 mm .

B.1.1.2 Irregular Small Opacities

Represented by the English letters s, t, u:

——s: The maximum width should not exceed 1.5 mm;

—— t : Width greater than 1.5 mm and not exceeding 3 mm;

——u: Width greater than 3 mm, not exceeding 10 mm.

B.1.1.3 Judgment and Recording Method

The determination of the shape and size of small shadows shall be based on the corresponding standard films.

When reading chest radiographs, the shape and size of small opacities should be recorded. If nearly all small opacities on the radiograph are of the same shape and size, their letter codes should be written above and below the slash, for example: p/p, s/s, etc. When there are small opacities of two or more shapes and sizes on the radiograph, the letter code for the predominant shape and size should be written above the slash, and the letter code for the secondary but still significant other type should be written below the slash, for example: p/q, s/p, q/t, etc.

B.1.2 Density

B.1.2.1 Four Major Levels of Classification

Density can be simply divided into four levels:

——Grade 0: No small opacities or very few, below the lower limit of Grade 1;

—— Level 1: A small amount of minor shadows;

——Level 2: Numerous small opacities;

——Level 3: Numerous small shadows.

B. 1. 2. 2 Twelve Minor Subdivision

The density of small opacities is a continuous gradient from sparse to dense. To objectively reflect this progression, each of the four major categories is further subdivided into three minor categories: 0/−,0/0,0/1 for Category 0; 1/0,1/1,1/2 for Category 1; 2/1,2/2,2/3 for Category 2; and 3/2,3/3,3/+ for Category 3. This classification aims to provide more detailed information, better reflect pathological conditions, and facilitate epidemiological research and medical surveillance.

B.1.2.3 Judgment and Recording Method

B.1.2.3.1 Judgment Principle

The determination of small shadow density should be based on the corresponding standard films, with the text serving only as an explanatory note.

B.1.2.3.2 Determination of Lung Zone Density

Based on the assessment of small opacity shapes, the density of small opacities in each lung zone is determined by comparing with the standard reference films of corresponding shapes, expressed in 12 minor grades. If the density of small opacities is essentially the same as that of the standard film, it can be recorded as 1/1, 2/2, or 3/3 respectively. If the density of small opacities is considered to be either one grade higher or lower than the standard film, both possibilities should be recorded, such as 2/1 or 2/3. The former indicates that the density is grade 2, but grade 1 should also be considered; the latter means the density is grade 2, but grade 3 should also be considered.

The principle for determining the profusion of lung zones is that the distribution of small opacities must cover at least two-thirds of the area of the zone.

B.1.2.3.3 Overall Density Determination

The overall profusion refers to the highest profusion of small opacities in any lung zone across the entire lung. It is a comprehensive assessment of the small opacity profusion throughout the lungs, based on the evaluation of profusion by individual lung zones, and is expressed using a 4-category scale.

B.1.2.3.4 Distribution Range Determination

The distribution range of small opacities refers to the number of lung zones with small opacities at profusion category 1 or higher.

B.2 Additional Symbols

Additional symbols include:

a) bu —— a pulmonary bulla;

b) ca —— lung cancer and pleural mesothelioma;

c) cn —— small shadow calcification;

d) cp —— cor pulmonale;

e) cv —— cavity;

f) ef —— pleural effusion;

g) em——emphysema;

h) es —— Eggshell calcification of lymph nodes;

i) ho ——honeycomb lung;

j) pc —— pleural calcification;

k) pt —— pleural thickening;

l) px —— pneumothorax;

m) rp —— rheumatoid pneumoconiosis;

n) tb —— active tuberculosis.

Appendix C (Normative Appendix) Chest Radiograph Quality and Quality Assessment

C.1 Chest X-ray Quality

C.1.1 Basic Requirements

C. 1.1.1 Should include both lung apices and costophrenic angles, with the sternoclavicular joints essentially symmetrical, and the shadows of the scapulae not overlapping the lung fields.

C. 1.1.2 The film number, date, and other markings should be placed above each shoulder respectively, arranged neatly, clearly visible, and not overlapping with the lung fields.

C. 1.1.3 The photo shall be free from artifacts, light leaks, contamination, scratches, water stains, and extraneous images.

C.1.2 Display of Anatomical Landmarks

C. 1.2.1 The lung markings on both sides are clear and sharply marginated, extending to the outer zones of the lung fields.

C. 1.2.2 The imaging of the cardiac border and diaphragmatic surface is sharp.

C. 1.2.3 Both lateral chest walls are well visualized from the lung apices to the costophrenic angles.

C. 1.2.4 The contours of the trachea, carina, and both main bronchi are visible, and the thoracic vertebral contours can also be displayed.

C. 1.2.5 Pulmonary markings in the retrocardiac region may be visible.

C. 1.2.6 The right dome of the diaphragm is generally located at the level of the tenth posterior rib.

C.1.3 Optical Density

C. 1.3.1 The maximum optical density in the upper and middle lung fields should be between 1.45 and 1.75.

C. 1.3.2 The optical density below the diaphragm is less than 0.28 on high-kilovoltage chest radiographs, and less than 0.30 on DR chest radiographs.

C. 1.3.3 The optical density of the direct exposure area is greater than 2.50.

C.2 Chest X-ray Quality Grading

C.2.1 Grade A Film (Excellent)

Fully meets the quality requirements for chest X-rays.

C.2.2 Grade B Film (Good Film)

Does not fully meet the quality requirements for chest X-rays, but has not yet degraded to grade C film.

C.2.3 Grade C Films (Poor Quality)

The following conditions classify as Grade C films and cannot be used for the initial diagnosis of pneumoconiosis:

a) The sum of defective areas that do not fully meet the basic requirements for chest radiograph quality and affect diagnosis ranges between half a lung zone and one lung zone;

b) The lung markings on both sides are not sufficiently clear and sharp, or localized lung markings are blurred, with the total area of affected regions impacting diagnosis ranging between half a lung zone to one lung zone;

c) The lateral chest wall from the lung apices to the costophrenic angles is poorly visualized, the tracheal outline is indistinct, and the lung markings in the retrocardiac region are difficult to discern;

d) Insufficient inspiration, with the right diaphragmatic dome positioned at or above the level of the eighth posterior rib;

e) The photo is too dark, with the highest optical density in the upper and middle lung zones between 1.85~1.90; or the photo is too bright, with the highest optical density in the upper and middle lung zones between 1.30~1.40; or the gray fog density is high, with the sub-diaphragm optical density between 0.40~0.50; or the direct exposure area optical density is between 2.20~2.30.

C.2.4 Garde D Films (Rejects)

Chest radiographs that do not meet the quality standards of Grade C are classified as Grade D and cannot be used for the diagnosis of pneumoconiosis.

Appendix D (Normative Appendix) X-ray Diagnostic Criteria for Pneumoconiosis

D.1 Relationship Between Standard Plates and Standard Provisions

The standard radiographs are an integral part of the diagnostic criteria for pneumoconiosis, primarily illustrating X-ray imaging changes that are difficult to describe in words. Therefore, the assessment of various X -ray imaging alterations in pneumoconiosis should be based on the standard radiographs, with the textual descriptions serving only as supplementary explanations.

D.2 Principles for the Preparation of Standard Sheets

The small shadow morphology and density expression are accurate and easy to use.

D.3 Composition and Content of Standard Specimens

The standard set consists of 7 composite films and 19 full-lung films. The composite films illustrate various shapes, sizes of small opacity profusion, and pleural plaques in different locations. The small opacity profusion composite films are arranged according to the midpoint of each profusion category, namely 0/0, 1/1, 2/2, 3/3. The full-lung films primarily demonstrate the relationship between small opacity profusion and distribution range across different stages of pneumoconiosis, as well as large opacities. Except for the chest radiographs specified as digital radiography in the standard set description, the rest are conventional high-kilovoltage chest radiographs.

D.4 Application of Standard Films

When reading chest X-rays for the diagnosis and staging of pneumoconiosis, especially when determining the shape, size, and profusion of small opacities, it is essential to compare them with the corresponding standard reference films.

Standard radiographs of the entire lung for each stage of pneumoconiosis serve as references for diagnosis and staging.

Appendix E (Normative Appendix) Technical Requirements for High Kilovoltage Chest Radiography

E.1 Photography Equipment

E.1.1 X-ray Machine

The maximum tube voltage output shall not be less than 125kV, and the power shall not be less than 20kW.

E.1.2 X-ray Tube and Window Filtration

E.1.2.1 Rotating Anode.

E.1.2.2 The focal spot shall not exceed 1.2 mm.

E.1.2.3 Total Window Filtration 2.5 mm ~ 3.5 mm Aluminum Equivalent.

E.1.3 Grid

E.1.3.1 The grid density shall not be less than 40 lines /cm.

E.1.3.2 The raster ratio shall not be less than 10:1.

E.1.3.3 Grating Focal Length 1.8 m.

E.1.3.4 Specifications Match the Film.

E.1.4 Intensifying Screens and Cassettes

E.1.4.1 Medium-speed intensifying screens are generally used.

E.1.4.2 The intensifying screen is free from stains.

E.1.4.3 The resolution of intensifying screens shall be no less than 5 line pairs /mm ~ 6 line pairs /mm.

E.1.4.4 The intensifying screen and film are in close contact.

E.1.4.5 The cassette is light-tight.

E.1.5 X-ray Film

E.1.5.1 Generally, universal-type (manual display, machine display) films are used, with a preference for dedicated films suitable for chest radiography.

E.1.5.2 Blue Base Film.

E.1.5.3 Base fog $D_{\min} < 0.20$.

E.1.5.4 Specification: $356\ \mathrm{mm} \times 356\ \mathrm{mm} (14\mathrm{in} \times 14\mathrm{in})$ or $356\ \mathrm{mm} \times 432\ \mathrm{mm} (14\mathrm{in} \times 17\mathrm{in})$.

E.1.6 Power Supply

E.1.6.1 The power supply shall comply with the rated requirements of the X -ray machine.

E. 1.6.2 X -ray machines require independent power supply and should not share the power source with motor-driven electrical appliances.

E.1.6.3 The power supply voltage fluctuation range is between ±10%.

E.2 Photography Techniques

E.2.1 Preparation and Positioning Requirements

E.2.1.1 The subject should press their chest wall firmly against the imaging stand, with feet naturally apart, and rotate their arms inward to minimize the overlap of the scapulae with the lung fields.

E.2.1.2 The focus-to-film distance is 1.80 m.

E.2.1.3 Adjust the tube position with the central line at the level of the sixth thoracic vertebra.

E.2.1.4 Exposure should be performed during breath-hold after full inspiration.

E.2.1.5 Posteroanterior chest radiographs are the standard examination. Additional views such as lateral, oblique, tomography, or CT scans may be performed as needed for diagnosis and differential diagnosis.

E.2.2 Photography Conditions

E.2.2.1 Use 120kV ~ 140kV for chest radiography based on the specific conditions of the X-ray machine.

E.2.2.2 Determine the exposure based on chest thickness, typically using 2mAs ~ 8mAs, with an exposure time not exceeding 0.1s.

E.2.2.3 When taking photographs, adjust the exposure settings with reference to previous chest X-rays.

E.3 Darkroom Techniques

E.3.1 Darkroom

The darkroom must meet the work requirements

E.3.2 Manual Hand Washing

E.3.2.1 In principle, constant temperature timing is required, the temperature of the chemical solution should be controlled between 20°C ~ 25°C, and the development time should be 3 min ~ 5 min.

E.3.2.2 Fixation should be thorough, and rinsing under running water must be complete.

E.3.2.3 Qualified dedicated safety lamps shall be used.

E.3.2.4 Replace the developer and fixer solutions in a timely manner.

E.4 Automatic Film Processor

To ensure the quality of chest X-rays, automatic film processors should be used whenever possible, and the operating procedures required by the automatic film processor must be strictly followed.

Appendix F (Normative Appendix) Technical Requirements for Digital Chest Radiography

F.1 Equipment Requirements

F.1.1 High-Frequency Inverter High-Voltage Generator: Maximum output power ≥20kW, inverter frequency ≥20kHz, output voltage 40kV~150kV.

F.1.2 Rotating Anode Tube: Nominal Focal Spot Values: Small focus ≤ 0.6; Large focus ≤ 1.3.

F.1.3 Upright Bucky Stand with Grid, Automatic Exposure Control (AEC), and Detector Field.

F.1.4 Flat Panel Detector: Effective detection area $\geq 365\ \text{mm} \times 365\ \text{mm}(14\text{in} \times 14\text{in})$, pixel size $\leq 200\mu\text{m}$; pixel matrix $\geq 2048 \times 2048$.

F.1.5 Grid: For tube voltages between $90\text{kV} \sim 125\text{kV}$, select a grid ratio of $10{:}1 \sim 15{:}1$, with a grid density of 34 lines/cm ~ 80 lines/cm.

F.2 Photography Requirements

F. 2.1 Photographic Position: Posteroanterior (PA) chest standing position. The subject should press the chest wall firmly against the imaging stand, with feet naturally apart and arms internally rotated to minimize overlap of the scapulae with the lung fields.

F.2.2 The source image distance (SID) is $180\ \text{cm}$.

F.2.3 Use a small focus.

F. 2.4 Adjust the tube position with the central line at the level of the sixth thoracic vertebra.

F.2.5 Use automatic exposure control (manual exposure may be used under special circumstances).

F.2.6 Photographic voltage: 100 kV~125 kV, exposure time: < 100 ms.

F.2.7 Exposure should be performed during breath-hold after full inspiration.

F.2.8 Protective Shielding: Standard Protection.

F.3 Image Processing

F.3.1 Before photography, it is advisable to set image processing parameters according to the quality requirements of pneumoconiosis chest radiographs.

F.3.2 Image processing should be performed prior to generating DICOM (Digital Imaging and Communications in Medicine) format image files. Image processing on DICOM format image files is not permitted.

F.3.3 Noise reduction, edge enhancement, and other image processing techniques should not be used.

F.3.4 The original image processing data should be retained.

F.4 DR Chest X-ray Medical Film Printing

F.4.1 Printing shall adhere to Quality Control (QC) procedures and comply with DICOM grayscale image display standards.

F.4.2 The printed chest radiograph should be life-size, matching the actual dimensions of the lungs, without any enlargement or reduction.

Appendix G (Normative Appendix) Requirements for Reading Radiographs in Pneumoconiosis Diagnosis

G.1 When reading films, it is generally recommended to sit down, with the position of the viewbox appropriately adjusted, typically placed at a distance of 25 cm (to facilitate observation of small shadows) to 50 cm (to facilitate observation of the entire chest film) in front of the reader.

G. 2 When reading films, one can observe and compare the dynamic changes in imaging by following the chronological order of chest radiographs.

G.3 When reading films, refer to standard films. Generally, place the chest film to be diagnosed in the center of the light box, with the standard films for reference on both sides.

G.4 The viewing box should be at least a 3-panel light box, preferably a 5-panel one. The minimum brightness of the viewing box should not be less than 3000 cd, with a brightness uniformity (brightness difference) of less than 15%.

G.5 The reading room should remain quiet, with no direct external light shining on the viewing box. The reading speed may vary according to personal habits, but a break should be taken approximately every 1 h ~ 1.5 h to maintain the reader’s visual acuity and mental clarity for optimal discernment.

“Diagnosis of Occupational Pneumoconiosis” (GBZ70-2015) Amendment No. 1

Appendix F.1.4 Flat Panel Detector: Effective detection area ≥ 365 mm × 365 mm (14 in × 14 in), ……

Change to:

Appendix F.1.4 Flat Panel Detector: Effective detection area ≥ 356 × 356 mm (14 in × 14 in), ……
